# Hippocampal Metabolic Alterations in Amyotrophic Lateral Sclerosis: A Magnetic Resonance Spectroscopy Study

**DOI:** 10.3390/life13020571

**Published:** 2023-02-17

**Authors:** Foteini Christidi, Georgios D. Argyropoulos, Efstratios Karavasilis, Georgios Velonakis, Vasiliki Zouvelou, Panagiotis Kourtesis, Varvara Pantoleon, Ee Ling Tan, Ariadne Daponte, Stavroula Aristeidou, Sofia Xirou, Panagiotis Ferentinos, Ioannis Evdokimidis, Michail Rentzos, Ioannis Seimenis, Peter Bede

**Affiliations:** 1 Medical Physics Laboratory, School of Medicine, National and Kapodistrian University of Athens, 115 27 Athens, Greece; 2 First Department of Neurology, Aeginition Hospital, School of Medicine, National and Kapodistrian University of Athens, 115 27 Athens, Greece; 3 Second Department of Psychiatry, Attikon General University Hospital, School of Medicine, National and Kapodistrian University of Athens, 124 62 Athens, Greece; 4 School of Medicine, Democritus University of Thrace, 681 00 Alexandroupolis, Greece; 5 Radiology Research Unit, School of Medicine, National and Kapodistrian University of Athens, 115 27 Athens, Greece; 6 Department of Psychology, National and Kapodistrian University of Athens, 157 84 Athens, Greece; 7 Department of Psychology, University of Edinburgh, Edinburgh EH8 9YL, UK; 8 Computational Neuroimaging Group, Trinity College, D02 R590 Dublin, Ireland; 9 Department of Neurology, St Jame’s Hospital, D08 NHY1 Dublin, Ireland

**Keywords:** amyotrophic lateral sclerosis, hippocampus, memory, magnetic resonance spectroscopy, metabolites, grey matter, white matter, tractography, perforant pathway

## Abstract

Background: Magnetic resonance spectroscopy (MRS) in amyotrophic lateral sclerosis (ALS) has been overwhelmingly applied to motor regions to date and our understanding of frontotemporal metabolic signatures is relatively limited. The association between metabolic alterations and cognitive performance in also poorly characterised. Material and Methods: In a multimodal, prospective pilot study, the structural, metabolic, and diffusivity profile of the hippocampus was systematically evaluated in patients with ALS. Patients underwent careful clinical and neurocognitive assessments. All patients were non-demented and exhibited normal memory performance. 1H-MRS spectra of the right and left hippocampi were acquired at 3.0T to determine the concentration of a panel of metabolites. The imaging protocol also included high-resolution T1-weighted structural imaging for subsequent hippocampal grey matter (GM) analyses and diffusion tensor imaging (DTI) for the tractographic evaluation of the integrity of the hippocampal perforant pathway zone (PPZ). Results: ALS patients exhibited higher hippocampal tNAA, tNAA/tCr and tCho bilaterally, despite the absence of volumetric and PPZ diffusivity differences between the two groups. Furthermore, superior memory performance was associated with higher hippocampal tNAA/tCr bilaterally. Both longer symptom duration and greater functional disability correlated with higher tCho levels. Conclusion: Hippocampal 1H-MRS may not only contribute to a better academic understanding of extra-motor disease burden in ALS, but given its sensitive correlations with validated clinical metrics, it may serve as practical biomarker for future clinical and clinical trial applications. Neuroimaging protocols in ALS should incorporate MRS in addition to standard structural, functional, and diffusion sequences.

## 1. Introduction

Amyotrophic lateral sclerosis (ALS) is a relentlessly progressive neurodegenerative condition with no effective disease-modifying therapies at present. Despite the insidious onset and the heterogeneity of initial presentations, most patients succumb to respiratory weakness [1]. Despite momentous advances in identifying genetic and epigenetic factors in its aetiology, the exact pathogenesis remains elusive in the majority of patients with sporadic disease. From a clinical standpoint, considerable differences exist in disease progression rates [1,2,3,4]. Although traditionally considered as a pure motor neuron disorder, it is now widely accepted that ALS can be associated with a variety of frontotemporal, extrapyramidal and cerebellar manifestations. Neuropsychological sequelae encompass multiple cognitive domains, including executive dysfunction, language deficits, memory impairment, and behavioural changes [5,6,7]. The ramifications of cognitive deficits are considerable with regards to multidisciplinary support needs. The implications of neuropsychological deficits on quality of life, caregiver burden, adherence to therapy, participation in clinical trials, engagement with health care professionals, and using assistive devices have been extensively studied. 

Computational neuroimaging has contributed significantly to our understanding of phenotype- and genotype-associated signatures and propagation patterns, and have helped to elucidate the anatomical substrate of clinical observations. Magnetic Resonance Imaging (MRI) and positron emission tomography (PET) studies have consistently confirmed extra-motor disease burden and have helped to validate staging systems proposed based on postmortem observations [5,8,9,10]. The practical utility of MRI stems from its biomarker potential, and both diagnostic and monitoring applications have been proposed. As pharmaceutical trial endpoints centres on survival and functional scores, the validation of imaging-based and laboratory markers is of significant interest [11,12]. 

Proton magnetic resonance spectroscopy (1H-MRS) allows the non-invasive quantification of focal metabolite concentrations in vivo [13,14]. The majority of MRS studies use proton MRS (1H-MRS) protocols which are easily implemented on most clinical MRI platforms [15]. 1H-MRS detects radiofrequency signals arising from hydrogen nuclear spins within tissue metabolites. These signals consist of metabolite-specific frequencies determined by the chemical environment of the hydrogen spins. Resulting MRS signals can be separated along a chemical spectrum, i.e., chemical shift dimension. The output spectrum is a plot of signal intensity, proportional to metabolite concentration, against resonance frequency. The latter is usually reported in field-independent units, i.e., parts per million of the proton frequency (ppm). The most common metabolites detected by 1H-MRS are lactate (Lac), a marker of anaerobic metabolism, lipids (Lip), a marker of anaerobic metabolism, alanine (Ala), an amino acid involved in the citric acid cycle, N-acetyl-asparate (NAA), a marker of neuronal integrity, creatine (Cr), a marker of energy metabolism, choline (Cho), a marker of membrane integrity, myo-inositol (mIns), a glial marker, glutamate (Glu), an excitatory neurotransmitter, glutamine (Gln), and γ-aminobutyric acid (GABA), an intracellular neurotransmitter. The combination of Glu and Gln is usually referred to as Glx. The number of quantifiable metabolites depends on the magnetic field strength, choice of pulse sequence parameters, spectral resolution, signal to noise ratio (SNR), B0 field homogeneity, and radiofrequency coil used [16]. For example, GABA is relatively challenging to reliably quantify at clinical field strengths due to the overlap between its resonance and that of Cr and other macromolecules necessitating special techniques and typically higher field strengths [17].

Since the very first MRS studies on ALS in the early 1990s [18], less than 100 MRS studies have been published on ALS, which is considerably less than the myriad of structural and functional neuroimaging studies [19]. With very few exceptions [20], MRS studies on ALS are primarily cerebral studies. Most MRS studies are single voxel initiatives, and whole-brain multi-voxel protocols have only recently been applied to ALS cohorts. Since the publication of pioneering studies on ALS, MRS have been used to ascertain medication effects, evaluate presymptomatic changes in mutation carriers, assess progressive metabolic alterations longitudinally, and explore subcortical changes [19]. Published MRS studies on ALS overwhelmingly focus on motor regions (i.e., motor cortex, pyramidal tract) and metabolic alterations in frontotemporal areas are poorly characterised. The most commonly reported findings on ALS are reduced NAA or NAA/Cr, NAA/Cho, NAA/Cr + Cho ratios, increased mIns, and increased/reduced Glu [19]. The metabolic profile of motor areas suggests early degeneration, correlates well with motor disability, reflects the lateralization of symptom predominance, is associated with progression rate, and is thought to also capture medication effect [19,21]. In a landmark longitudinal study, Kalra and colleagues [22] detected increased NAA/Cr ratio in the primary motor cortex of ALS patients treated with Riluzole, establishing 1H-MRS as an academically and clinically useful imaging modality [19,21]. From a potential diagnostic perspective, MRS exhibits good discriminant power between patients and controls, particularly when combined with structural imaging data [23,24,25]. 

The clinical heterogeneity of ALS is compellingly supported by structural [26,27,28,29], diffusivity [27,29,30] and functional neuroimaging studies [31,32]) and have well identified neuroimaging patterns in line with postmortem descriptions [33,34]. However, there is a paucity of frontotemporal 1H-MRS studies [19]. These typically identify reduced NAA or NAA ratios in dorsolateral prefrontal [35,36], medial prefrontal [37,38], cingulate [39], thalamic [39,40] and basal ganglia [40] regions. Few 1H-MRS studies report neuropsychological measures, and they suggest an association between dorsolateral prefrontal metabolic changes and executive performance [35,36]. 

With the gradual recognition of frontotemporal dysfunction in ALS, imaging studies have increasingly turned their focus from the precentral gyrus to orbitofrontal [41,42], subcortical [43], thalamic [28], dorsolateral prefrontal [44], and superior temporal regions [45,46]. Neuropsychological deficits in ALS were initially thought to be dominated by executive deficits, hence the predominant interest in prefrontal cortex integrity. However, it is now increasingly recognised that memory impairment, language deficits, apathy, and deficits in social cognition [47] are also common manifestations of ALS, and accordingly imaging studies have started to explore the integrity of associated anatomical regions in more detail. Consistent with postmortem observations [48,49,50,51], the hippocampus is now recognised as an important structure involved in ALS [52], with disease-specific radiological signatures [30,53], progressive structural degeneration [54], and functional changes [55] in line with clinical decline [56]. Postmortem studies unequivocally demonstrate hippocampal involvement in the latter stages of ALS [57], and neuropathological alterations have also been observed in patients without overt dementia [58,59]. Similarly, in vivo neuroimaging and neuropsychological studies have consistently detected early memory dysfunction and hippocampal changes in ALS without dementia [52]. Notwithstanding the advances in hippocampal imaging in ALS, striking gaps can be identified in the literature. The vast majority of the studies rely solely on structural data, limiting the analyses to volumetric, morphometric and vertex-wise analyses [60,61,62]. There is a paucity of studies examining the integrity of hippocampal white matter projections, but metabolic alterations have not characterised to date. The integrative evaluation of structural, metabolic, and diffusivity metrics may help to establish the chronology of pathological processes, contrast the detection sensitivity of various imaging metrics, and assess the biomarker potential of radiological indices [63,64,65,66]. 

Accordingly, our objective is to systematically characterise the structural, diffusivity, and metabolic profile of the hippocampus in ALS using a multimodal protocol. Our hypothesis is that hippocampal 1H-MRS captures a unique signature in ALS patients without dementia.

## 2. Materials and Methods

### 2.1. Ethics Approval

This prospective neuroimaging study has been approved by the institutional review board of Aeginition University Hospital (AΔA, ΨΔ4846Ψ8Ν2-Γ9Φ/06-11-2020) and all participants provided informed consent before inclusion. 

### 2.2. Participants

Sixteen patients with ALS diagnosed according to the revised El Escorial criteria [67] have been enrolled. A group of 14 healthy controls (HC) with a comparable demographic profile was also included. Exclusion criteria for study participation included comorbid neurological conditions, established psychiatric illness, psychoactive medications that may affect memory performance, and a clinical diagnosis of frontotemporal dementia, and contraindications for MRI examination. 

### 2.3. Cognitive Assessment 

A single clinical neuropsychologist administered a standardised battery of cognitive tests in the exact same order on the day of the MR image acquisition. All psychometric measures were standardized in Greek language and normative values established in the local population. Cognitive assessment included the Edinburgh Cognitive and Behavioural Amyotrophic Lateral Sclerosis (ECAS) [68,69], a brief multidimensional scale that assesses executive functions (e.g., working memory, social cognition, inhibition, alternation), verbal fluency, memory (immediate recall, delayed recall, retention), language, and visuospatial functions (e.g., simple and composite visuospatial organization and planning). Patients’ ECAS scores were interpreted based on local normative data [68]. For the current study, all ECAS memory scores (total, immediate recall, delayed recall, delayed recall index, and recognition) were explored in further analyses. 

### 2.4. MRI Data Acquisition

All participants underwent standardized brain imaging protocol on a 3 Tesla Achieva-Tx Philips (Best, The Netherlands) manufactured MRI system equipped with an 8-channel receive head coil. Each participant’s head was positioned in the scanner by placing foam wedges on both head sides to immobilize their head in the coil.

We obtained high resolution anatomical images applying a 3D T1-weighted turbo field echo sequence [inversion time: 1200 ms, repetition time (TR): 9.9 ms, echo time (TE): 3.7 ms, flip angle: 7o, voxel-size: 1 × 1 × 1 mm, matrix size: 244 × 240, 170 slices]. WM microstructural integrity was estimated using diffusion tensor imaging (DTI) technique, acquired with an axial single-shot spin-echo echo-planar imaging sequence with 30 diffusion encoding directions and the following parameters: TR: 7299 ms, TE: 68 ms, flip angle: 90o, field of view: 256 × 256 mm, voxel size: 2 × 2 × 2 mm, 70 slices. A T2 weighted combined with fluid attenuated inversion recovery technique was acquired to identify hyperintense brain lesions (TR: 11,000 ms, TI: 2800 ms, TE: 125 ms, acquisition matrix: 384 × 186, slice thickness 4 mm). 

**Figure 1:**
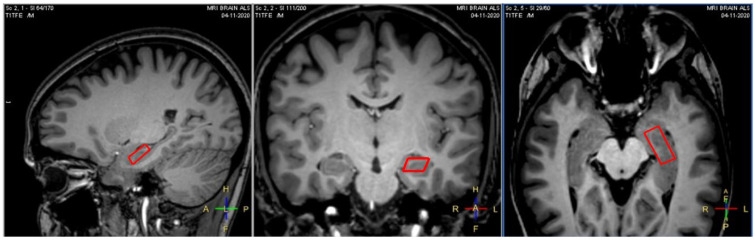
1H-MRS voxel position [9 (RL) × 23 (AP) × 9 (FH) mm] in left hippocampus (data from a healthy control).

Single-voxel point resolved spectroscopy (PRESS) pulse sequence was used for spectrum acquisition with TR = 2000 ms, TE = 35 ms, and NSA = 256 combined with water suppression chemically selective saturation pulses to suppress the water signal. Water’s curve full width at half maximum (FWHM), displayed in the MRI monitor during the acquisition preparation phase, was used as the first quality indicator to estimate the local field homogeneity. The cutoff value was set at 15 Hz. The MRS voxel (Figure 1) was oriented along the anterior-posterior hippocampal axis and its dimensions were: (1) left-right, 9 mm; (2) anterior-posterior, 23 mm; (3) superior-inferior, 9 mm. Of note, a healthy participant was scanned three times with a time interval of two weeks between each exam to address issues related to sequence optimization and voxel placement consistency (reproducibility). 

### 2.5. MRI Data Analysis

#### 2.5.1. MRS Spectroscopy

**Figure 2:**
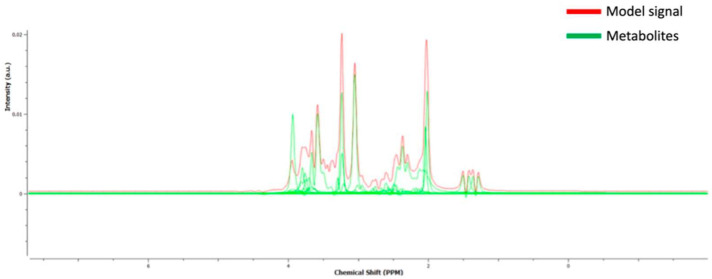
A representative spectrum with the fitted peaks (Tarquin software, version 4.3.10).

Raw spectroscopy data were exported from the scanner and metabolites’ concentrations (mM) were quantified using TARQUIN (version 4.3.10) [70]. Based on widely adopted spectral quality criteria [71], we excluded six participants (four ALS and two HC) from further analyses. These exclusion criteria are based on the TARQUIN quality calculated parameters of FWHM < 0.15 ppm, SNR > 5, and measure of fit quality < 2.5 for quantification reliability and spectral quality [71]. Additional comparisons on quantification reliability and spectral quality parameters were performed between ALS and HC for both hippocampi to check any systematic quantification bias (Supplementary Table S1). Voxel water signal was used as a reference signal to estimate the metabolites’ concentration. We used water as a reference for metabolite concentration estimation, since it is the only choice in Tarquin software. Furthermore, to the best of our knowledge, most research teams use water as the reference metabolite when they estimate the metabolites’ absolute concentrations [72,73,74]. In addition, normalization to another metabolite may dissimulate comparable changes in the two metabolites. During pre-processing steps, spectroscopic data were corrected for eddy currents and frequency drifting by 1H NAA Cr Cho internal basis as reference signal. For reliable spectroscopy analysis, accurate baseline modelling is crucial especially at short time echoes [75]. Since we considered lipids as metabolites of no particular interest, the lipid filter was set to on and the internal basis to 1H brain + Glutathione (Glth) + no Lip/MM to decrease the risk of noise modelling (baseline overfitting) [70]. All other parameters were left the same as default. In the calculation of absolute concentrations, we applied the correction factor used previously [76] to account for the different metabolites’ distribution in the GM and WM tissue including in the MRS voxel. GM and WM fractions were estimated using previously published Matlab code [66]. Metabolites’ concentrations were exported in mM units. A representative spectrum with the fitted peaks (Tarquin software) is provided in Figure 2. Individual metabolite fitting for the metabolites used in the current study is presented in Supplementary Figure S1. 

The following metabolites were included in further analyses: tNAA, tCho, tCr, Glu, and Ins. Based on previous motor and a few extra-motor MRS studies [19], we also calculated the following ratios using the metabolites’ absolute values: tNAA/tCho, tNAA/tCr, tCho/tCr, Glu/tNAA, Glu/tCho, Glu/tCr, Ins/tNAA, Ins/tCho, Ins/tCr.

#### 2.5.2. Hippocampal GM Analysis

**Figure 3:**
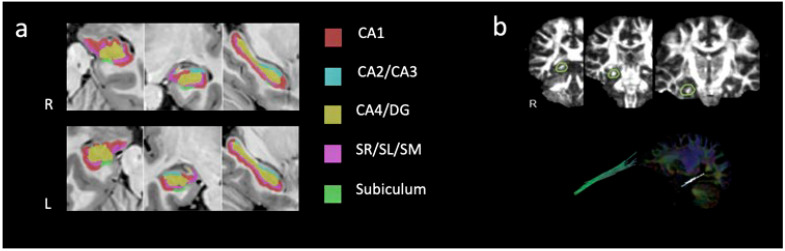
(**a**) Hippocampal segmentation and resulting subfields in axial, coronal, and sagittal views and (**b**) the tractographic reconstruction of the right perforant pathway zone using multiple-ROI analysis with its respective ROI gates on fractional anisotropy maps.

Anatomical data from HR_3DT1w were processed using the volBrain system (http://volbrain.upv.es (accessed on 9 September 2022)), which provides automatic segmentation of different brain structures from anatomical T1-weighted images. Automatic segmentation of the hippocampus was performed with HIPS [77] and a combination of non-linear registration and patch-based label fusion was included [78]. The following subfields (Figure 3a) are automatically segmented according to the protocol proposed by Winterburn [79]: CA1; CA2/CA3; CA4/dentate gyrus (CA4/GD); strata radiatum/lacunosum/moleculare (SR/SL/SM); subiculum. The segmented maps from the up-sampled T1-weighted images are then down-sampled to fit the MNI space resolution. Absolute values (measured in cm^3^) and relative values (measured in relation to total intracranial volume-TICV) are provided for each subfield, separately for the left and the right hippocampus. 

#### 2.5.3. Hippocampal WM Tractography

DTI analysis was conducted using the Brainance MD (Advantis Medical Imaging, Eindhoven, the Netherlands). Motion and eddy current correction with the registration tool available in the MR scanner and co-registration protocol with Brainance MD were conducted before tractography analysis. The reconstruction of the hippocampal PPZ was based on multiple region-of-interest (ROI) tractography (Figure 3b) which is described in detail elsewhere [30,80]. The following DTI parameters were automatically extracted and included in further analyses: fractional anisotropy (FA), axial diffusivity (AD), and radial diffusivity (RD). Intra- and inter-rater reliability were assessed for all tracts in each participant and showed high intra-class correlation (ICC > 0.8). 

### 2.6. Statistical Analyses

Assumptions of normality were examined using the Kolmogorov-Smirnov test. Because of the sample size of our study, non-parametric tests were applied. Comparisons between ALS and HC groups on MRS data were performed using the Mann-Whitney U test. To further enhance the accuracy of the estimates of significance, Monte Carlo simulation was utilised, sampling of 30,000 combinations, and computation of the 99% confidence interval (C.I.) of the *p*-value. Effect sizes for non-parametric comparisons were also calculated based on the recommended formula (r = Z/√N) and interpreted as follows: r = 0.1 (small), r = 0.3 (medium), r = 0.5 (large) [81]. To test the effect of diagnosis on volumetric and tractographic data, we ran Quade non-parametric analyses of covariance using MRI data (subfields absolute volumes and FA, AD and RD metrics as dependent variables), group (ALS and HC) as independent variable, and total intracranial volume (only for the volumetric comparisons), age, and sex as covariates. Correlations analyses (Spearman’s rho) were conducted within the ALS group between MRS data and patients’ clinical (ALSFRS-R, disease duration, progression rate) and memory scores (ECAS total memory, ECAS immediate recall, ECAS delayed recall, ECAS delayed recall index, ECAS recognition). The significance level was set at *p* < 0.05. All analyses were conducted using the IBM SPSS package (v. 28.0). 

## 3. Results

### 3.1. Sample Characteristics

The final sample consisted of 12 patients with ALS and 12 HC (Table 1). Groups were matched for age, sex, education, and total MMSE score. None of the patients scored below the normal cut-off values in ECAS-total score [mean (sd) = 109.83 ± 5.29, min-max = 103–120, cut-off: ≤93/136], ECAS-ALS Specific [mean (sd) = 78.50 ± 4.66, min-max = 72–85, cut-off: ≤68/100], ECAS-ALS Non-Specific [mean (sd) = 31.17 ± 3.04, min-max = 25–35, cut-off: ≤23/36] and ECAS-memory domain [mean (sd) = 19.25 ± 2.86, min-max = 14–23, cut-off: ≤12/24] based on available normative data for the local population [68]. 

### 3.2. The MRS Spectroscopy Profile of Hippocampus in ALS

**Figure 4:**
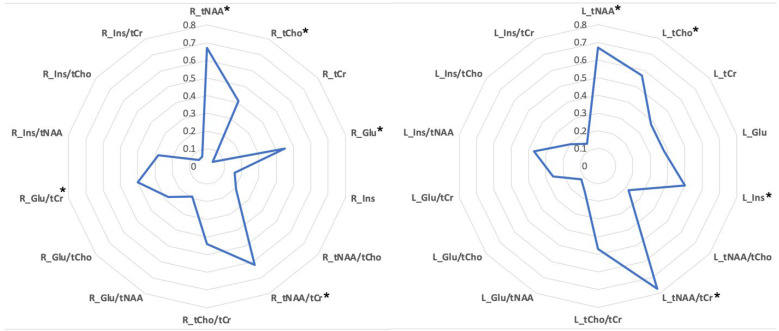
Cohen’s effect size chart of metabolites’ alterations of ALS patients compared to HC in right and left hippocampus. Metabolites with significant between-group differences (Mann-Whitney U test, Monte Carlo method, sampling n = 30,000) are further highlighted with *.

Compared to HC, ALS patients showed higher tNAA, tNAA/tCr and tCho bilaterally, higher Glu and Glu/tCr in right hippocampus, and higher Ins in left hippocampus (Table 2). The magnitude of between-group differences (Cohen’s |r| effect size) is presented in Figure 4. Large effect sizes were found for tNAA (bilaterally), tNAA/tCr (left), tCho (left) and Ins (left) while medium effect sizes were found for tCho (right), tCr (left), Glu (bilaterally), tCho/tCr (bilaterally), Glu/tCr (right), and Ins/tNAA (left).

### 3.3. The GM Profile of Hippocampus in ALS

**Figure 5:**
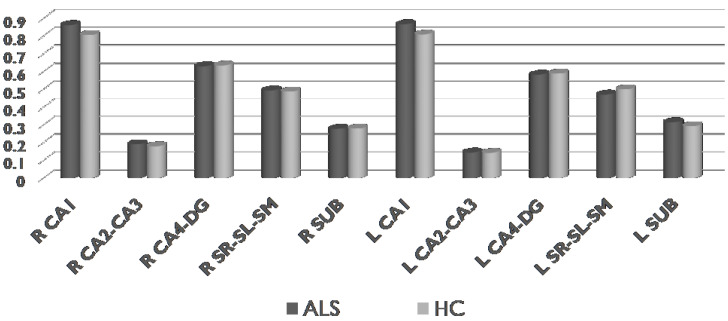
Comparisons of the volumes of the hippocampal subfields in ALS and HC (Mann-Whitney U-test, Monte Carlo method, sampling n = 30,000). ALS = amyotrophic lateral sclerosis, HC = healthy controls, R = right, L = left, CA = cornu ammonis, DG = dentate gyrus, SR-SL-SM = strata radiatum-lacunosum-moleculare, SUB = subiculum.

We did not find any significant differences (*p* > 0.05) between ALS and HC neither in total hippocampal volume nor in subfield volumes of right and left hippocampus (Figure 5). 

### 3.4. The WM Profile of Hippocampus in ALS 

**Figure 6:**
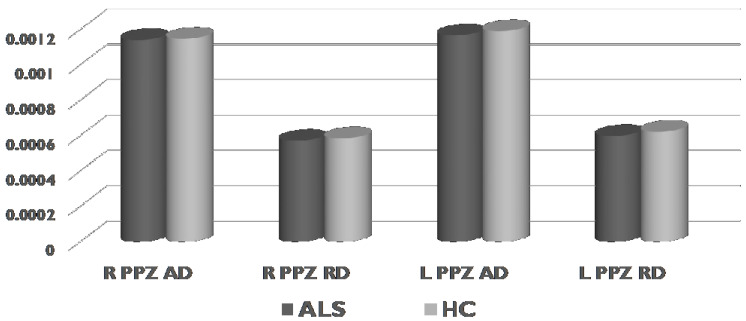
Comparisons of the diffusivity values (AD and RD) of the hippocampal PPZ in ALS and HC (Mann-Whitney U-test, Monte Carlo method, sampling n = 30,000). AD = axial diffusivity, RD = radial diffusivity, PPZ = perforant pathway zone, ALS = amyotrophic lateral sclerosis, HC = healthy controls, R = right, L = left.

We did not find any significant differences (*p* > 0.05) between ALS and HC neither in FA nor in diffusivity indices (AD and RD, Figure 6) of left and right PPZ. 

### 3.5. Correlations between MRS Spectroscopy Data and Clinical and Memory-Related Data within ALS 

Correlation analyses between MRS data with significant between-group differences and clinical and memory data were conducted within the ALS group. 

ALS functional status (i.e., ALSFRS-R) was negatively associated with left hippocampal tCho (r = −0.65, *p* = 0.023) and positively associated with left hippocampal tNAA/tCr (r = 0.70, *p* = 0.011), i.e., better functional status (higher ALSFRS-R) was associated with lower tCho and higher tNAA/tCr. Disease duration was positively associated with right hippocampal tCho (r = 0.73, *p* = 0.007) and negatively associated with right hippocampal Glu/tCr (r = −0.73, *p* = 0.007) and left hippocampal Ins (r = −0.70, *p* = 0.011). With regards to memory scores, we found a positive association between ECAS Delayed Recognition and left hippocampal Ins (r = 0.70, *p* = 0.011) and right hippocampal tNAA/tCr (r = 0.58, *p* = 0.048), as well as ECAS Delayed Recall Index and left hippocampal tNAA/tCr (r = 0.66, *p* = 0.020).

## 4. Discussion

Our study detected increased NAA and Cho levels in the hippocampi of non-demented ALS patients bilaterally and an association between memory measures and hippocampal NAA/Cr ratios. It is noteworthy that these patients do not exhibit hippocampal atrophy at the time of their scan and no integrity alterations in their PPZ DTI metrics. 

The vast majority of MRS studies report reduced NAA levels in the precentral gyrus, often in conjunction with reduced focal grey matter density or thickness [5,19,82]. Increased NAA levels are seldom reported in ALS, which is most likely due to the combination of the choice of region-of-interest (primarily motor regions) and the stage of the disease (often late-stage study inclusion). There is also a notion that focal MRS changes may precede the structural degeneration of a specific anatomical region based on which MRS has been coined as an early harbinger of impending pathology. Our cohort does not exhibit hippocampal atrophy, shows no evidence of degenerative change in their hippocampal WM projections, and their neurocognitive scores are normal. Of note, there were no differences neither on the total hippocampal GM and subfield GM volume nor on the GM and WM volume within the MRS voxel between ALS patients and HC. Increased NAA levels identified may suggest the functional recruitment of this brain region and could potentially be interpreted as a compensatory process. Choline, a cell membrane marker, is also higher in our ALS cohort. Increased choline levels are classically associated with increased cellular membrane turnover and linked to processes such as demyelination, inflammation, and gliosis [83]. It is noteworthy, however, that high choline peaks are also observed in the context of active membrane synthesis such as in paediatric populations [84]. 

Cognitive reserve [85,86,87], and to a lesser extent motor reserve [88], have been increasingly studied in ALS and there is a suggestion that premorbid functional activity may translate to a certain resilience against functional decline or lead to a delay in symptom onset. Most imaging studies to date have struggled to compellingly demonstrate the effect of cognitive reserve in ALS, but research efforts are ongoing. From a motor perspective, however, it is clear that by the time the diagnosis is established, widespread degenerative change can be readily captured radiologically, including anatomical regions of functional motor domains which are still unaffected. [89] The observation that motor areas already show degenerative change without clinical manifestations [89] and the body of literature on presymptomatic motor cortex or corticospinal tract changes in asymptomatic mutation carriers suggest that anatomical structures can accumulate a considerable disease burden before clinical symptoms ensure. Simply put, there seems to be a fair amount of physiological redundancy or functional back-up, and certain disease burden thresholds need to be reached before symptoms actually manifest. 

The divergence of disease burden and functional disability is relatively well known to ALS researchers [90], and even though direct clinico-radiological correlations are often requested, it is increasingly recognised that no simplistic associations exist between radiological integrity measures and clinical disability. The modifiers of clinical features at a given level of disease burden are likely to include a spectrum of genetic, developmental, education, motor training and other “performance reserve” factors. Notwithstanding these caveats, we did identify a positive correlation between Delayed Recognition on ECAS and tNAA/tCr, as well as between the Delayed Recall Index and tNAA/tCr. Most of the correlations included left hippocampal metabolites in line with the lateralization hypothesis and the involvement of left hemisphere/hippocampus in verbal memory processes [91,92]. Previous ALS studies have also reported significant associations between verbal memory tests (i.e., immediate and delayed story recall scores) and bilateral hippocampal GM volumes [93]. Of note, functional imaging studies highlight that the stage of memory processing is also related to the lateralization, with encoding producing left-lateralized patterns of activations and retrieval producing right-lateralized patterns of activations [94]. The association between focal metabolite ratios and structure mediated functional domains support the practical utility and detection sensitivity of MRS in ALS. Cognitive performance has been repeatedly linked to structural measures of mesial temporal lobe integrity [30,61,95,96], but the detection sensitivity of spectroscopic measures with regards to clinical correlates have not been previously explored. The striking association between symptom duration and right hippocampal tCho is of particular interest. As a marker of cell membrane turnover, increasing tCho levels over time may represent progressive focal inflammatory change. This interpretation is further supported by the negative association between ALSFRS-R and left hippocampal tCho. It may be that as the disease progresses and functional disability ensues, focal membrane breakdown and microglial activity manifests in increased local choline concentrations. 

These findings underscore the clinical utility of MRS in a progressive neurodegenerative condition like ALS; it offers a much-needed metabolic insight into molecular processes that may not be readily captured by the volumetric and morphometric methods typically applied to structural datasets. The limitations of structural imaging in ALS are seldom enunciated with sufficient candour, and these are particularly apparent when structural imaging is used in isolation without supporting fMRI and DTI data. Often, motor cortex changes are not detected by structural pipelines alone, and efforts to discriminate PLS and ALS patients based on T1-weighted and DTI data alone in machine-learning frameworks have been disappointing [97,98]. Accurate individual patient classification into diagnostic, phenotypic, or prognostic categories is an emerging field of ALS [98,99,100] and a multitude of promising initiatives have been reported using either clinical variables alone [101,102,103], imaging metrics [62,97,104], or both [105]. Careful feature selection is indispensable for effective MRI-based machine-learning strategies, and most existing studies use solely structural and DTI data [106,107]. The addition of MRS variables may enhance the diagnostic performance of recently proposed machine learning frameworks. Another trend of “big data” interrogation in ALS is the implementation of various clustering approaches to unravel inherent, naturally occurring sub-groups or patient cohorts with distinctive characteristics. A number of recent imaging studies have confirmed the existence of radiological sub-phenotypes using cluster analyses of connectomics [108], functional [109], or structural [110] raw datasets. The inclusion of MRS data into similar clustering pipelines may further help to untangle the heterogeneity of ALS and identify subcohorts with distinctive radiological characteristics. 

Similar to our study, MRS metrics have consistently captured the substrate of clinical disability, medication effects, and progressive clinical decline [19,22,82], suggesting that it is a worthy addition to both academic and clinical protocols in ALS. Pharmacological trials of ALS continue to primarily rely on clinical metrics and wet biomarkers [95,111], and the few multi-site imaging initiatives [112,113,114] rely on structural, functional, and DTI data alone. There is now ample evidence that MRS is both sensitive to detect focal changes and exhibit good correlations with relevant clinical metrics and therefore should be incorporated in both future single-centre and multi-centre studies. The challenges of voxel placement consistency and ROI selection are likely to be overcome by emerging whole-brain, multi-voxel techniques that have already been successfully piloted in ALS [115,116,117]. 

Our study is not without limitations. The sample size of this pilot study is relatively small, and while it demonstrates the feasibility and clinical potential of hippocampal spectroscopy, it lacks the statistical power and the inclusion of different ALS phenotypes and genotypes. We did not apply multiple comparisons, but we used Monte Carlo simulation and presented the exact *p*-values for all statistical analyses and the effect sizes for the magnitude of difference in between-group comparisons. As this is a cross-sectional study, the ability of MRS to track progressive temporal lobe pathology and verify its prognostic value remains to be established by future longitudinal studies. In addition, the ability to resolve glutamate alone using short echo PRESS at 3T is questionable. The increasing use of ultra-high field strength MRI scanners is expected to facilitate the reliable separation of glutamate signal from the glutamine signal [118,119,120]. The employment of a control region might have strengthened hippocampal findings, although increasing evidence from advanced neuroimaging literature suggests that regions previously known as unaffected are also affected and thorough clinical, neurophysiological and neuropsychological investigation may further provide anatomo-clinical associations (e.g., [121,122,123]). Notwithstanding these limitations, our study demonstrates the utility of MRS to examine non-motor brain regions in ALS and the metabolic substrate of specific cognitive domains. Our results indicate that MRS is a clinically useful imaging modality in ALS and can be readily implemented for the assessment of extra–motor brain regions. Larger studies, longitudinal study designs, and whole-brain, multi-voxel approaches are needed for the comprehensive characterisation of metabolic alterations in ALS and to clarify the comparative detection sensitivity of MRS compared to structural, functional, and diffusivity data derived metrics. Based on our preliminary findings, additional studies are warranted to further evaluate the associations between hippocampal metabolites’ profile and memory function, by employing a thorough and detailed examination of memory function (i.e., specific tests for verbal and visual memory).

## 5. Conclusions

Our pilot data suggest that hippocampal NAA/Cr metabolite ratios show associations with memory performance, and symptom duration correlates with choline levels. Our results demonstrate the utility of extra-motor MRS in ALS, even in a cohort without overt cognitive impairment. MRS is a sensitive and easy-to-implement imaging modality with a considerable pragmatic potential for both academic and clinical applications.

## Figures and Tables

**Table 1:** The demographic and clinical profile of study participants.

	ALS (*n* = 12)	HC (*n* = 12)	Statistical Difference
Age (years)	59.83 ± 10.53	52.92 ± 9.37	0.103
Sex (M/F)	7/5	5/7	0.414
Education (years)	14.08 ± 2.81	14.17 ± 2.59	0.940
Handedness (Rt/Lt)	12/0	12/0	-
MMSE	28.25 ± 1.22	28.50 ± 1.17	0.613
Disease duration from symptom onset (m)	25.33 ± 22.74	-	-
ALSFRS-R	38.25 ± 7.89	-	-

Note. ALS = healthy controls; HC = healthy controls; M/F = male/female; Rt/Lt = right/left; MMSE = Mini-Mental State Examination; m = months; ALSFRS-R = Amyotrophic Lateral Sclerosis Functional Rating Scale-Revised. Between-group comparisons were performed using t-test for independent samples (age, education, and MMSE) and χ2 (gender distribution).

**Table 2:** The metabolite concentration profile of ALS and HC in left and right hippocampus.

	ALS (*n* = 12)	HC (*n* = 12)	*p*-Value
Right hippocampus			
tNAA	8.71 ± 1.29	6.25 ± 1.34	**<0.001**
tCho	2.33 ± 0.66	1.95 ± 0.44	**0.044**
tCr	8.58 ± 1.34	8.85 ± 2.58	0.886
Glu	9.14 ± 3.20	5.86 ± 3.50	**0.028**
Ins	11.32 ± 2.37	10.01 ± 3.89	0.433
tNAA/tCho	4.04 ± 1.32	3.36 ± 0.97	0.311
tNAA/tCr	1.02 ± 0.12	0.76 ± 0.25	**0.001**
tCho/tCr	0.27 ± 0.06	0.23 ± 0.05	**0.033**
Glu/tNAA	1.07 ± 0.40	0.88 ± 0.47	0.371
Glu/tCho	4.49 ± 2.52	3.11 ± 1.90	0.175
Glu/tCr	1.09 ± 0.42	0.69 ± 0.40	0.051
Ins/tNAA	1.35 ± 0.49	1.65 ± 0.65	0.178
Ins/tCho	5.91 ± 4.71	5.57 ± 3.01	0.799
Ins/tCr	1.37 ± 0.45	1.24 ± 0.58	0.799
Left hippocampus			
tNAA	11.45 ± 4.31	5.57 ± 2.22	**<0.001**
tCho	3.00 ±1.50	1.82 ± 0.78	**0.004**
tCr	9.34 ± 4.03	7.35 ± 1.82	0.066
Glu	11.26 ± 9.24	5.82 ± 2.59	0.065
Ins	12.34 ± 4.04	8.52 ± 1.96	**0.013**
tNAA/tCho	5.03 ± 4.12	3.29 ± 1.31	0.284
tNAA/tCr	1.27 ± 0.17	0.78 ± 0.31	**<0.001**
tCho/tCr	0.31 ± 0.08	0.24 ± 0.07	**0.020**
Glu/tNAA	0.90 ± 0.54	1.27 ± 0.88	0.431
Glu/tCho	3.35 ± 2.15	3.74 ± 1.94	0.578
Glu/tCr	1.09 ± 0.66	0.83 ± 0.37	0.214
Ins/tNAA	1.37 ± 1.21	2.10 ± 2.00	0.075
Ins/tCho	11.26 ± 24.98	5.66 ± 2.84	0.342
Ins/tCr	1.87 ± 2.06	1.23 ± 0.39	0.514

Note. Metabolites’ absolute concentrations are presented in mM. Bold *p*-values denote significant differences between ALS and HC groups based on Mann-Whitney U test (Monte Carlo method, sampling n = 30,000). ALS = healthy controls; HC = healthy controls; tNAA = total N-acetyl-asparate; tCho = total Choline; tCr = total Creatine; Glu = glutamate; Ins = inositol; mM = millimolar.

## Data Availability

The data presented in this study are available on request from the corresponding author.

## Supplementary Materials

The following supporting information can be downloaded at: https://www.mdpi.com/article/10.3390/life13020571/s1. Table S1: Quantification reliability and spectral quality parameters of ALS and HC for the right and left hippocampal; Figure S1: Individual metabolite fitting (Tarquin software, version 4.3.10) for metabolites used in the current study (tNAA, tCho, tCr, Glu, Ins). The red line represents model signal, while the green lines denote individual metabolites. tNAA = total NAA; tNAA = N-acetylaspartate (NAA) + N-acetylaspartateglutame (NAAG); tCho = total Cho; tCho = Glycerophosphocholine (GPC) + Phosphocholine (PCH); tCr = total Cr; tCr = creatine (CR) + Phospho-creatine (PCR); GLU = glutamate; INS = inositol.
