# Supplementary material for: Hippocampal Metabolic Alterations in Amyotrophic Lateral Sclerosis: A Magnetic Resonance Spectroscopy Study
Source: Life (Basel). 2023 Feb 17;13(2):571. doi: 10.3390/life13020571 (PMC9965919; doi:10.3390/life13020571)
Supplement: Supplementary file 1 [file life-2095310-supplementary.pdf › life-2095310-supplementary/life-2095310-supplementary.pdf]

Table S1. Quantification reliability and spectral quality parameters of ALS and HC for the right and left hippocampal

	ALS	HC	Statistics (p-value)
**Right hippocampus**			
**FWHM (ppm)**	0.075 ± 0.024	0.058 ± 0.011	0.105
**SNR**	8.455 ± 1.478	7.156 ± 1.030	0.261
**Q**	1.368 ± 0.113	1.213 ± 0.121	0.069
**Left hippocampus**			
**FWHM (ppm)**	0.065 ± 0.016	0.062 ± 0.019	0.662
**SNR**	8.425 ± 2.338	6.787 ± 2.126	0.093
**Q**	1.349 ± 0.137	1.267 ± 0.096	0.106

Note. ALS = amyotrophic lateral sclerosis; HC = healthy controls; FWHM = full width at half maximum; SNR = signal to noise ratio; Q = measure of fit quality; ppm = parts per million of the proton frequency

Figure S1. Individual metabolite fitting (Tarquin software, version 4.3.10) for metabolites used in the current study (tNAA, tCho, tCr, Glu, Ins). The red line represents model signal, while the green lines denote individual metabolites. tNAA = total NAA; tNAA = N-acetylaspartate (NAA) + N-acetylaspartateglutame (NAAG); tCho = total Cho; tCho = Glycerophosphocholine (GPC) + Phosphocholine (PCH); tCr = total Cr; tCr = creatine (CR) + Phospho-creatine (PCR); GLU = glutamate; INS = inositol

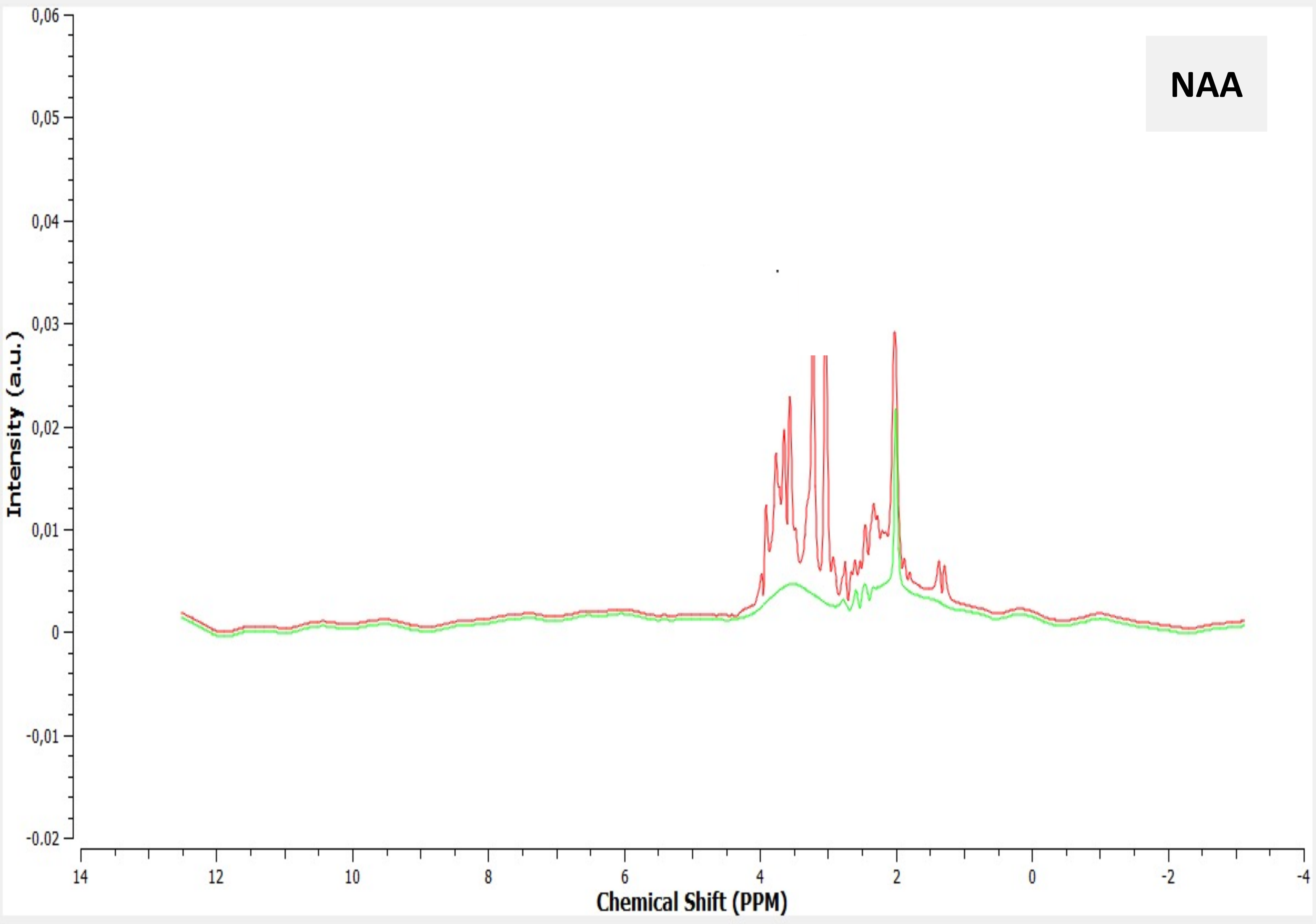
NAA
Intensity (a.u.)
0,06
0,05
0,04
0,03
0,02
0,01
0
-0,01
-0,02
14 12 10 8 6 4 2 0 -2 -4
Chemical Shift (PPM)

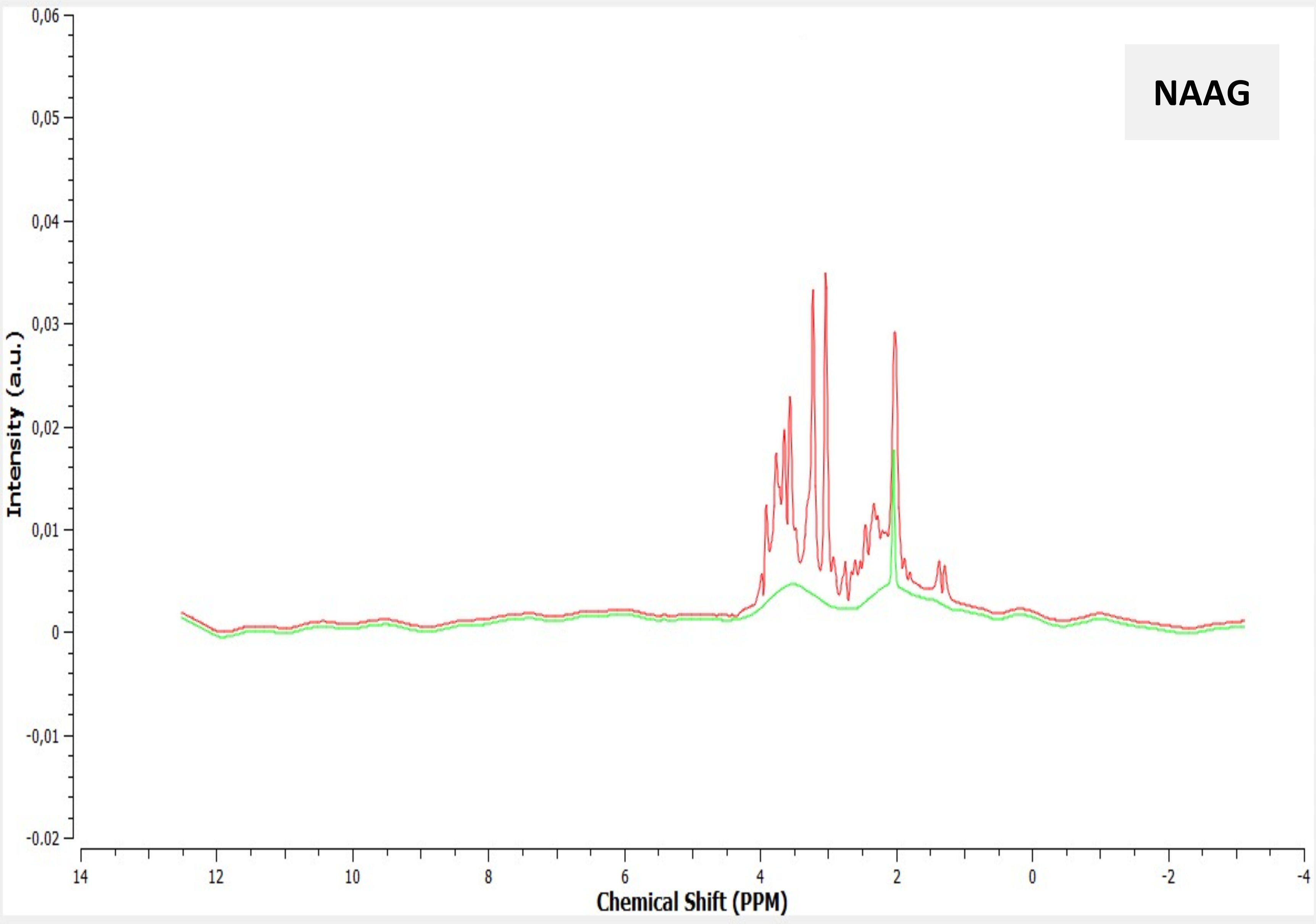
NAAG
Intensity (a.u.)
0,06
0,05
0,04
0,03
0,02
0,01
0
-0,01
-0,02
14 12 10 8 6 4 2 0 -2 -4
Chemical Shift (PPM)

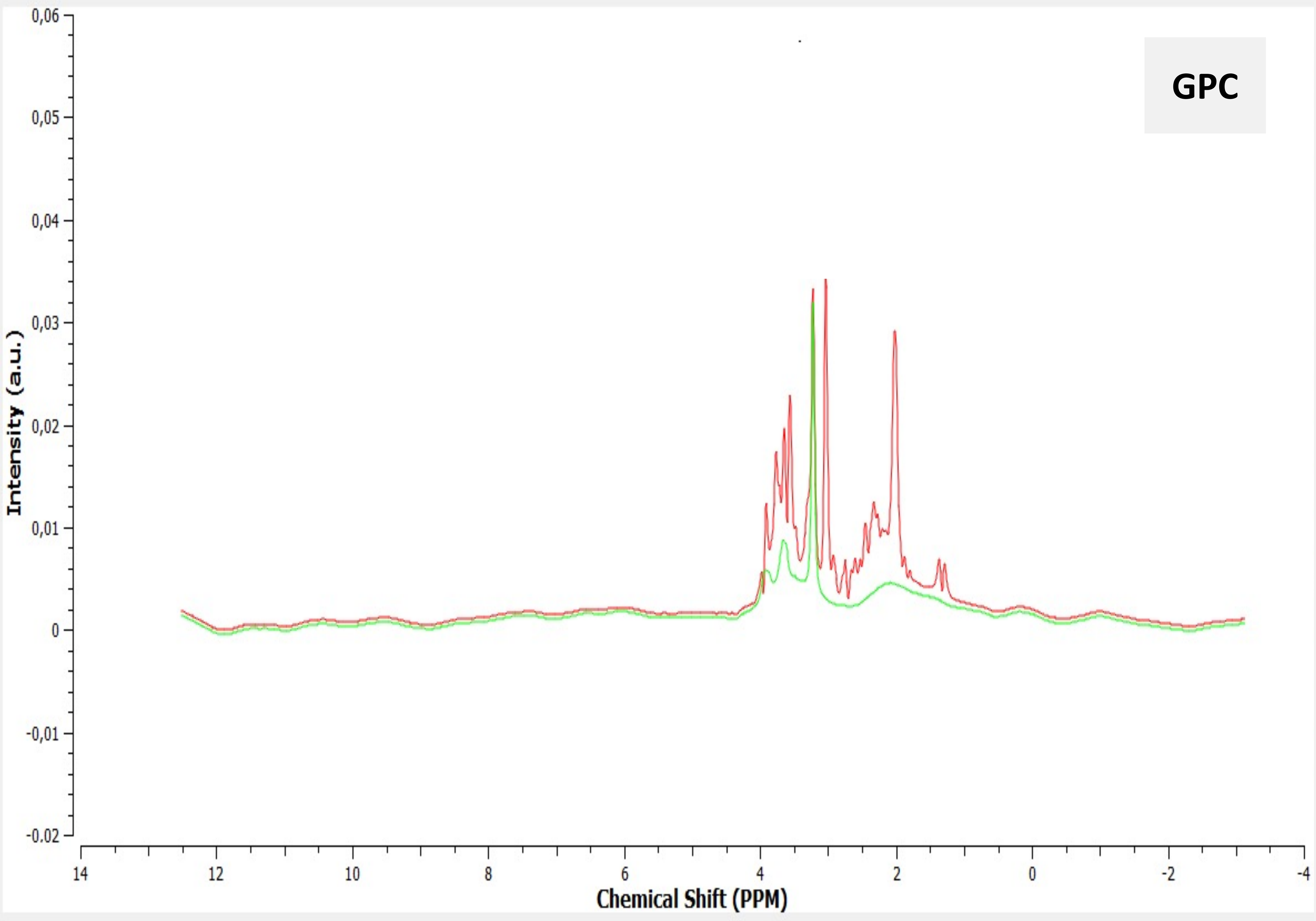
GPC
Intensity (a.u.)
0,06
0,05
0,04
0,03
0,02
0,01
0
-0,01
-0,02
14 12 10 8 6 4 2 0 -2 -4
Chemical Shift (PPM)

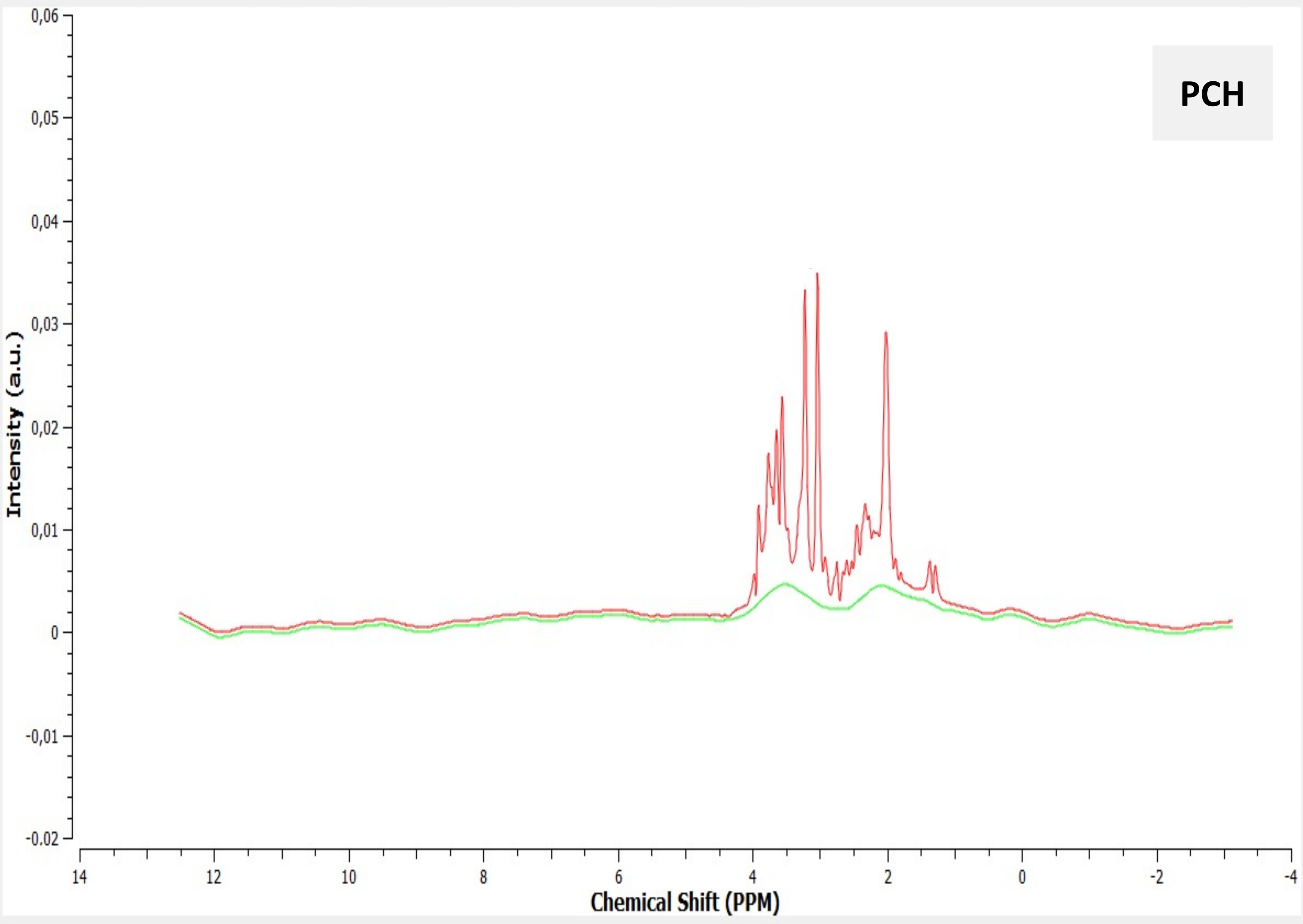
PCH
Intensity (a.u.)
0,06
0,05
0,04
0,03
0,02
0,01
0
-0,01
-0,02
14 12 10 8 6 4 2 0 -2 -4
Chemical Shift (PPM)

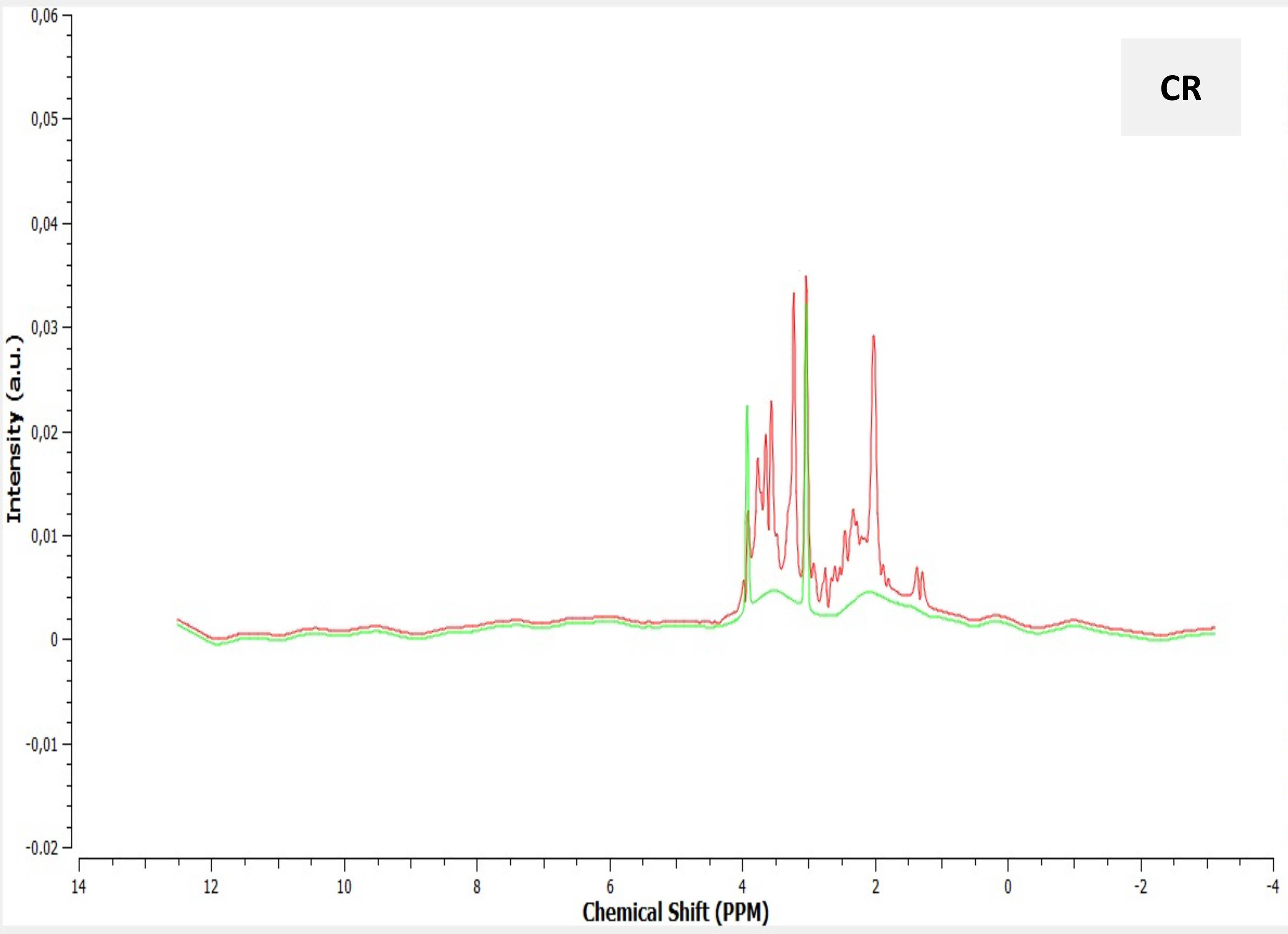
CR
Intensity (a.u.)
0,06
0,05
0,04
0,03
0,02
0,01
0
-0,01
-0,02
14 12 10 8 6 4 2 0 -2 -4
Chemical Shift (PPM)

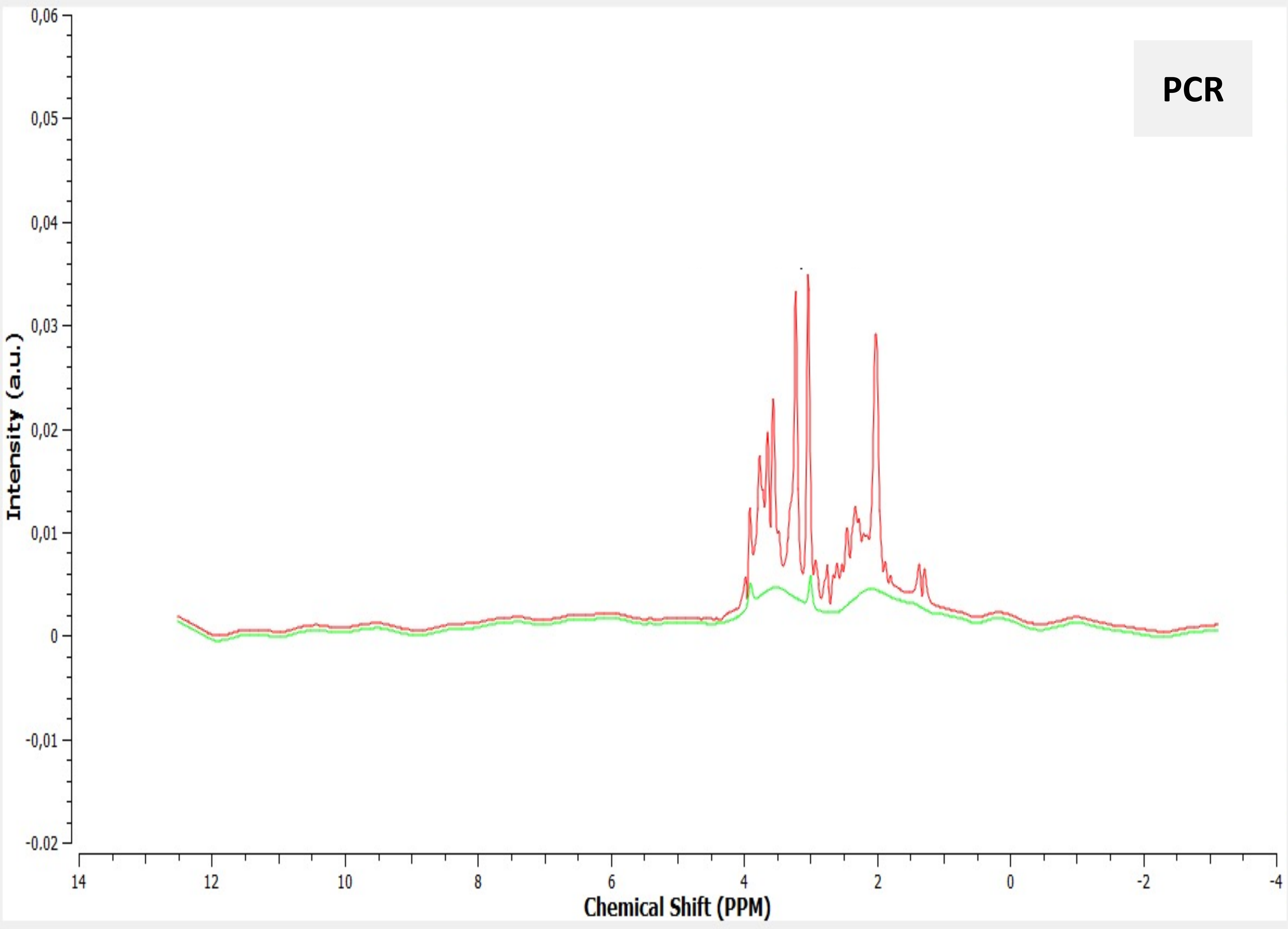
PCR
Intensity (a.u.)
0,06
0,05
0,04
0,03
0,02
0,01
0
-0,01
-0,02
14 12 10 8 6 4 2 0 -2 -4
Chemical Shift (PPM)

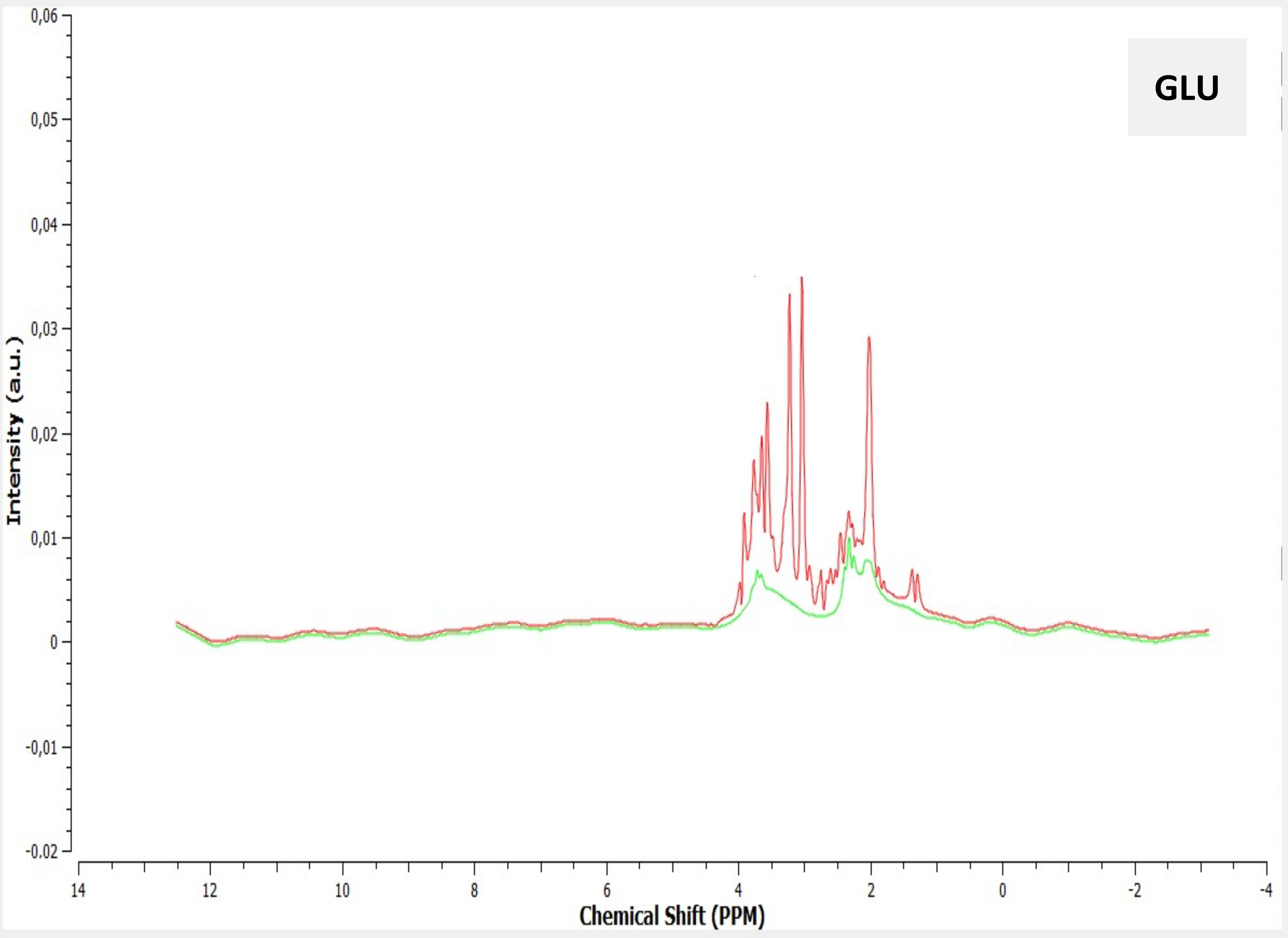
GLU
Intensity (a.u.)
0,06
0,05
0,04
0,03
0,02
0,01
0
-0,01
-0,02
14 12 10 8 6 4 2 0 -2 -4
Chemical Shift (PPM)

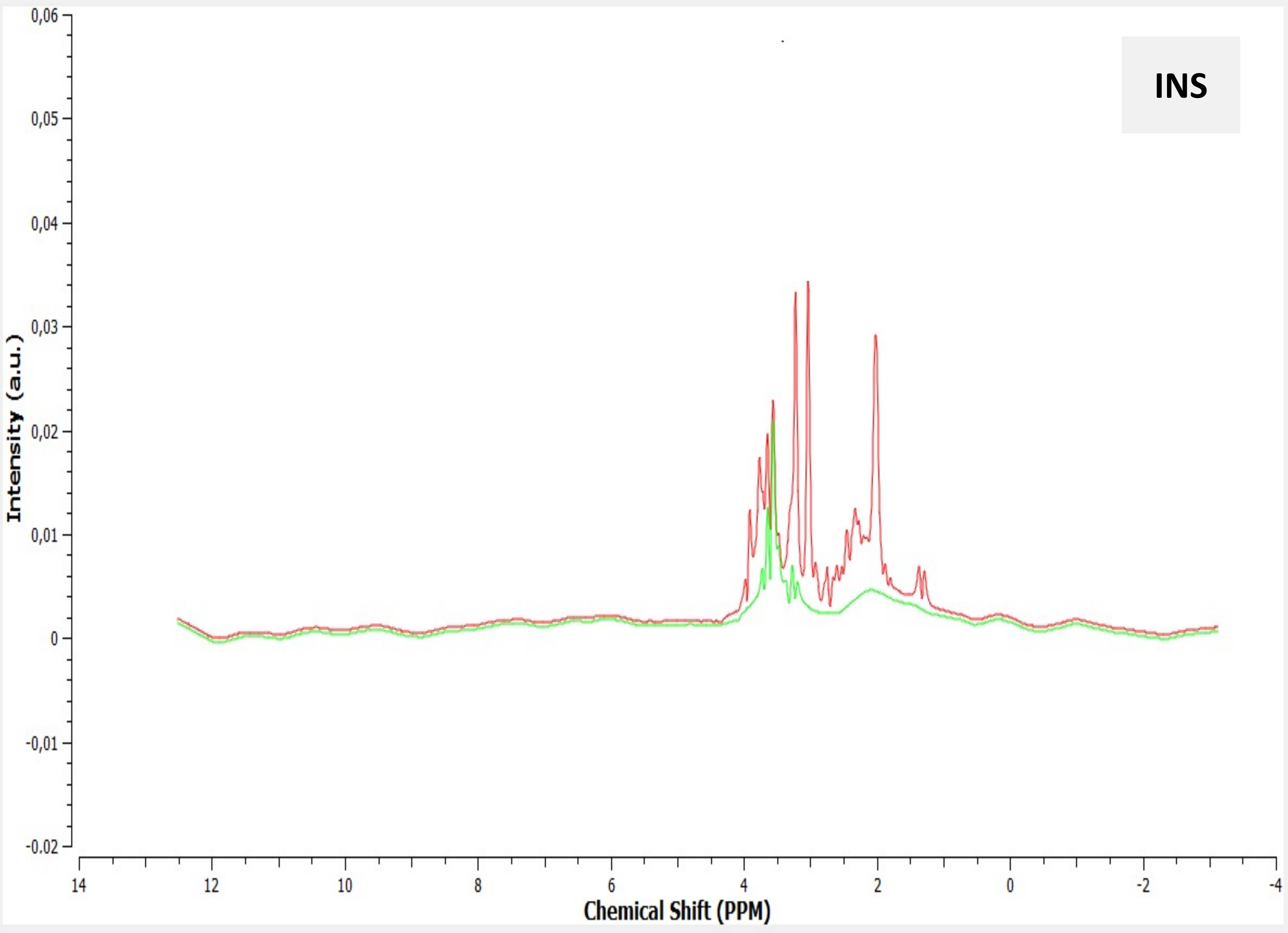
INS
Intensity (a.u.)
0,06
0,05
0,04
0,03
0,02
0,01
0
-0,01
-0,02
14 12 10 8 6 4 2 0 -2 -4
Chemical Shift (PPM)
